# In-migration, customary land tenure, and complexity: exploring the relationship between changing land tenure norms and differentiated migrant livelihoods in Brong Ahafo, Ghana

**DOI:** 10.1007/s11111-017-0277-z

**Published:** 2017-04-21

**Authors:** Jon Sward

**Affiliations:** Sussex Centre for Migration Research, University of Sussex, Falmer, Brighton, East Sussex BN1 9QN UK

**Keywords:** Migration, Land tenure, Adaptation, Poverty, Rural livelihoods

## Abstract

This article focuses on the relationship between in-migration from Northern Ghana and changing land tenure norms in Ghana’s central “transition zone” in Brong Ahafo Region. Using the complex adaptive systems (CAS) theoretical framework, it theorizes this relationship as part of a wider set of “co-evolving” social and environmental conditions across Brong Ahafo. It presents new qualitative research findings which show differentiated livelihood trajectories for Northern Ghanaian migrant farmers in Brong Ahafo in three case study sites in different districts and links these to migrants’ diverse land tenure arrangements under customary tenure regimes in Brong Ahafo. I argue that differentiated outcomes for migrants at rural destinations have implications for the extent to which out-migration from environmentally marginal regions such as Northern Ghana can be viewed as a form of “adaptation” to environmental change.

## Introduction: conceptualizing migration, land tenure, and wider rural transformations as part of a “complex adaptive system”

In recent years, there has been an explosion of literature on whether migration out of marginal environmental locations such as Northern Ghana^1^ can be considered a form of adaptation to environmental change (Foresight 2011; Afifi et al. 2016; McLeman and Hunter 2010). However, as Morrissey (2012) points out, there has been insufficient attention paid to how land tenure interfaces with the environment and migration debate in terms how it may influence migration decisions and to understanding why customary tenure regimes function as they do. This article explores the relationship between in-migration and customary land tenure in Brong Ahafo Region, Ghana, based on qualitative research conducted at three comparative case study sites in 2014. As noted by Van der Geest et al. (2010) and Moller-Jensen and Knudsen (2008), internal migration from Northern Ghana to agricultural areas in western and central Ghana constitutes a key secondary internal migration pattern in the country, alongside rural–urban migration to cities such as Accra, Kumasi, and other growing urban centers. In the case of Brong Ahafo, migrants to rural areas typically engage in rain-fed agriculture of commercial food crops, including maize, cassava, yam, and groundnuts.

With no formal claim to farmland under Brong Ahafo’s customary land tenure system—which generally holds that locals with “first-comer” status have customary access rights to land (Afikorah-Danquah 1997)—migrant farmers from Northern Ghana typically rely on rental or sharecropping agreements which they secure through locals (typically village chiefs or local families). However, the terms of migrants’ access to land can vary according to factors such as migrants’ time of arrival at destination, their varying social capital, which may smooth access in some cases, and varying demands for, and quality of, farmland in different parts of Brong Ahafo. This article considers the ways in which migrants from Northern Ghana interact with customary land tenure institutions in Brong Ahafo and the implications of this in terms of migrants’ livelihood outcomes. The article positions the interplay between in-migration and land tenure in Brong Ahafo Region within the complex adaptive systems (CAS) framework (cf. Rammel et al. 2007) in order to consider this in-migration as part of a wider set of co-evolving relationships made up of social and environmental components. This framework posits that actors’ behavior – or agency – is mediated by specific conditions, which in turn results in particular “feedbacks” emerging. These feedbacks may alter the behavior of actors, thus leading to a change in the underlying conditions of the wider system.

Based on empirical findings in Brong Ahafo, the article introduces a typology of migrant farmer livelihood trajectories, ranging from “highly adaptive” to “non-adaptive”. It explores how different livelihood trajectories inform Northern Ghanaian migrants’ interactions within Brong Ahafo’s customary tenure system, as well as their implications for migration’s potential to reduce poverty or increase resilience of migrants’ “left-behind” kin in Northern Ghana. Migrants’ differentiated livelihood trajectories affect their agency, helping to determine their adaptive pathways with respect to the wider “social–ecological system” (c.f. Oliver-Smith 2009). Following recent work by Ramalingam (2013) and Burns and Worsely (2015), who have highlighted the relevance of research on complexity for re-thinking wider development efforts, I argue that using this theoretical lens to analyze migration from Northern Ghana to Brong Ahafo sheds light on the relationship between in-migration and the local social–ecological system, illuminating how social and environmental factors at destination affect migration’s potential to serve as an adaptation out of environmentally marginal areas such as Northern Ghana.

This is significant, as in the specific case under investigation here migrants have moved out of a context of relative poverty in Northern Ghana, with farming opportunities in Brong Ahafo representing a comparatively attractive livelihood option to many Northern Ghanaian migrants. As Nyantakyi-Frimpong and Bezner-Kerr (2015: 41) observe, Northern Ghana remains a development “paradox on virtually every front”, with 80% of the population engaged in agriculture, food insecurity and child malnourishment affecting high levels of the population, and poverty rates typically two-to-three times the national average. Moreover, Marchetta (2013) and Awumbila et al. (2015) have both demonstrated that internal migration is a livelihood strategy more often pursued by less well-off households in Northern Ghana. Van der Geest (2011a) has argued that this is in part related to a structural scarcity of good-quality arable land in Northern Ghana, which he cites as a key reason for out-migration to rural destinations elsewhere in Ghana, including Brong Ahafo and Western Region.^2^ Another key aspect of this migration is the fact that such destinations have a more favorable rainfall regime than Northern Ghana: For example, Brong Ahafo has two rainy seasons per year, with peak rains occurring from May–June and September–October, which constitute the major and minor growing seasons respectively (see Owusu and Waylen 2013), compared to just one annual rainy season in Northern Ghana.

The article is structured as follows: the next section explores the existing debates about customary land tenure administration in Sub-Saharan Africa, and relates these to the specific case of in-migration from Northern Ghana to Brong Ahafo. The subsequent section presents qualitative research findings from three case study sites in Brong Ahafo, discussing the research methodology used for the study as well as explaining the distinct findings on the localized relationship between in-migration and land tenure across the three sites. Following this, the paper introduces a typology of migrant livelihood trajectories at the three sites, and provides an analysis of how land tenure norms relate to livelihood trajectories for different migrant actors. Finally, the paper concludes by considering the need to take into account environmental conditions at destinations – in this case changing land tenure norms at rural destinations in West Africa – within the wider debate about whether migration can serve as an adaptation to environmental change.

## Customary land tenure and in-migration: Key features of Ghana’s rural “complex adaptive system”

In Ghana, customary land administration constitutes the primary institution governing land use in rural agricultural zones. As Ubink (2009: 52) notes, “the ‘customary’ dominates both property rights and allocational authority: 80% of land is regulated by customary law, with a decisive role for traditional authorities.” Traditional Authorities, or chiefs, are recognized as the “legitimate owners over the land and representatives of the rural people” (Amanor 2005, p. 117). However, the ultimate authority of chiefs over customary land runs parallel to the de facto ownership rights of local families, who often pass user rights to specific pieces of land down from generation to generation (see, for example, Afikorah-Danquah 1997).

Such overlapping claims to land represent a key feature of the relatively fluid nature of customary tenure in Ghana and elsewhere in sub-Saharan Africa. Lentz argues in this regard:

customary tenure… has never been as static or homogeneous as many policy makers and researchers have assumed. Even in pre-colonial times, and more so during colonial rule and after independence, indigenous tenure regimes were not coherent and stable systems of rules and beliefs, but contested pastiches of historically grounded arguments about property rights and access to land resources as well as to membership in the local political community (Lentz 2013, p. 8).

Relatedly, recent academic debate on customary land tenure in sub-Saharan Africa has focused on the links between membership in social networks, political processes, and access to land, with disagreement emerging over whether poor people are more likely to have access to land under customary tenure (cf. Berry 1993, p. 104; see also Toulmin and Quan 2000 and Toulmin et al. 2002) or whether customary land administration in fact exacerbates inequality, owing to stratifications within social networks and local power structures (see for example Daley and Hobley 2005; Juul and Lund 2002; Lund 2000; and Woodhouse 2003). One key area that this debate has focused on is the relationship between customary land administration and women’s access rights. In their influential critique of women’s access rights under customary land administration in sub-Saharan Africa, Whitehead and Tsikata (2003) note that customary tenure regimes can in fact exacerbate gender-based inequalities. They observe that, in the context of wider debates about the utility of the “customary” in ensuring equitable access to land for all land claimants, “…insufficient attention has been paid to power relations in the countryside, and the implications for social groups, including women, who are not well represented in local-level power structures” (Whitehead and Tsikata 2003, p. 67).

Migrants are another notable “marginal” group within customary tenure frameworks. As Whitehouse (2012) notes, there is a long history of interactions between locals and migrants, or “strangers,” across sub-Saharan Africa, as well as a literature stemming from this (see for example Adida 2014; Shack and Skinner 1979). Whitehouse (2012) argues that the stranger in Africa falls within Simmel’s (1950) elaboration of the term, which he sees as a paradoxical figure who is both part of and excluded from society, as is manifested in urban settings through the frequent establishment of ethnic enclaves. In contemporary rural Africa, meanwhile, the question of land tenure norms—as defined by autochthony based on who is perceived as part of the group and who is defined as a stranger—“is among the most crucial and controversial in African politics” (Bøås 2009, p. 20).

In the specific case of Ghana, the nature of migrant-host relations varies widely in different parts of the country and even within individual communities. In general, land scarcity has emerged in parts of Ghana in recent decades, which has affected how land is transferred among family members who have a customary claim to specific pieces of land, and this has had implications for migrant-host relations. As Amanor argues, in Ghana’s forest zone since the 1970s:

Increasing scarcity of land has hindered the transmission of land across generations, as well as the use of gifts of land within the family to build up family labor networks. … Increasing areas of family land are allocated as sharecrop arrangements to non-kin rather than being inherited by kin members (Amanor 2008, p. 72).

Not surprisingly, the nature of such arrangements has meant that migrants have been involved in land conflicts in some parts of Ghana in recent years. For example, Quan et al. (2008) observe that pilot land registration projects in Ghana’s Eastern and Western Regions created tensions over contested claims to land, including land allocated to migrants. Elsewhere, Lobnibe (2008) notes that in Brong Ahafo, village chiefs sometimes intentionally allocated contested land to migrants, with such arrangements intended to solidify their own underlying claims to these tracts. This is in line with Amanor and Pabi’s (2007) argument that under customary land administration in Brong Ahafo, where multiple parties may have potential claims to land, keeping land continuously occupied—via agreements with migrants or otherwise—is one way of expressing de facto land ownership.

With this background in mind, I theorize that land tenure arrangements for Northern Ghanaian migrants in Brong Ahafo reflect a wider “complex adaptive system”—composed of “co-evolving” and dynamic social and ecological conditions—including in-migration, land availability, and the emergence of more commercialized land rental markets. Amanor observed of Brong Ahafo in the 1990s that, “The area is one of the least densely populated in Ghana. Land values are not highly commoditized, unlike in other areas of the forest” (Amanor 1994, p. 34). The relative abundance of available farmland in Brong Ahafo is one factor that has encouraged in-migration of tenant farmers from Northern Ghana in recent decades, yet this process itself has begun to alter land’s availability and perceived value in different parts of the region. As shall be explored below, the terms of migrants’ access to land reflect a diversity of migrant-host relations. While many migrants enter seasonal rental or sharecropping arrangements with local families in order to access land, in other cases, tracts of land have been ceded to migrants via historical agreements with village chiefs, financial transactions, or—less commonly—intermarriage with local families. The diversity of these arrangements in turn affects the relative security of migrants’ land tenure, which I argue is a key aspect of their agency as actors within a wider “complex adaptive system,” framing how they interact with their “host” environs. In turn, such differentiated migrant agency has important implications for theorizing out-migration from Northern Ghana as a form of “adaptation” to environmental change.

## In-migration and evolving land tenure norms: comparative findings from three Northern Ghanaian migrant communities in Brong Ahafo

**Fig. 1:**
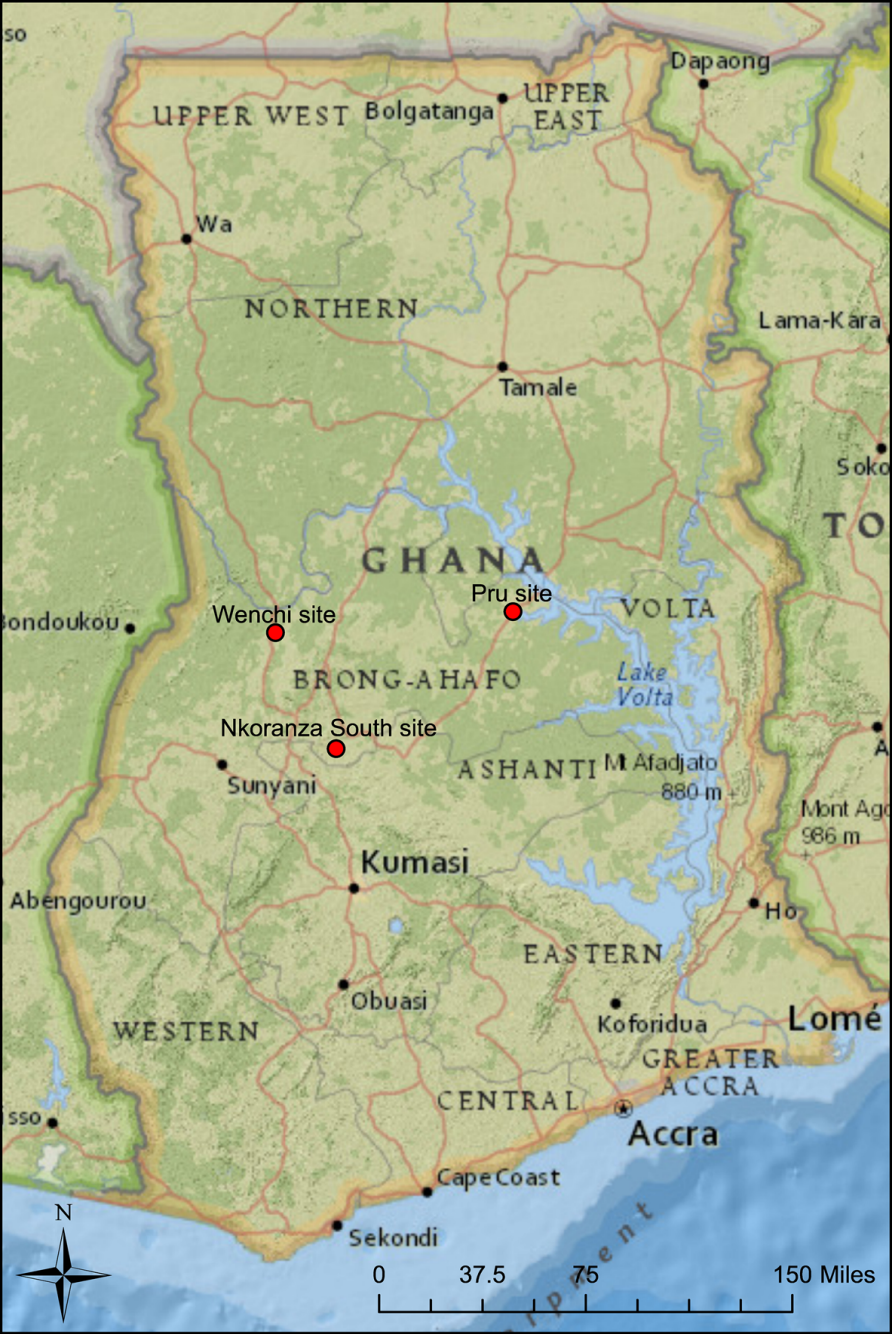
Fieldwork sites (locations approximate to protect research participant anonymity; created using ArcMap)

This section explores how tenure arrangements between migrants from Northern Ghana and their local hosts differed in the three case study communities where qualitative interview data was collected in 2014 (see Fig. 1). It also describes how land tenure practices concerning migrants had evolved over time in the three communities, per interview data. The section explains the methodology that was used for the qualitative research, before discussing land tenure norms for migrant farmers at the three sites. These qualitative insights help to illustrate the ways in which migrants’ land tenure arrangements vary across Brong Ahafo Region. I theorize these differences, via the complex adaptive systems (CAS) framework, as being rooted in differing social and agro-ecological conditions at the respective case study sites.

### Research methodology

As part of the research methodology, I identified three case study sites in different districts of Brong Ahafo Region—Nkoranza South, Wenchi Municipal, and Pru Districts. The sites consisted of migrant “settler” communities, referred to in Twi language (an Akan dialect and local lingua franca in Brong Ahafo), as *atukɔ tenafoɔ akuraa* (which literally translates as “refugee village”), where Northern Ghanaian migrants constituted the majority population. The sites were chosen due to *contrasting* key features, including differing local migration histories (each site attracted migrants from different origin areas of Northern Ghana and had different timescales of migration), distinct local land tenure norms, and varying agro-ecological conditions (including rainfall, soil quality, etc.). Additionally, the districts where the three field sites were located had varying population densities: According to the 2010 Ghana national census, Nkoranza South District had a population density of 109.3 persons/km^2^, while Wenchi Municipal District averaged 69.2 persons/km^2^ and Pru District had just 40.1 persons/km^2^. The selection of contrasting case study sites was undertaken in order to theorize in-migration as part of a wider “complex adaptive system,” with differing local social and environmental conditions at each community designed to tease out the heterogeneous ways in which migration forms part of ongoing changes in land’s availability and perceived value across Brong Ahafo Region.

The field sites were selected after a preliminary visit to Brong Ahafo in November 2013 where seven potential field sites were visited, with the research itself conducted between March and May 2014. Community access was gained through initial meetings with local chiefs secured through local gatekeepers, who gave permission for the research to take place in each instance. The research consisted primarily of individual, semi-structured interviews conducted in Twi, typically lasting between 45 and 90 min, which were translated during each interview by interpreters from the Nkoranza-based migration NGO, Scholars in Transit.^3^ While migrant research participants were not randomly selected to ensure that interviews were representative, care was taken to ensure that they did reflect a cross section of Northern Ghanaian migrant ethnic groups living in each community, as well as different age groupings, genders, and wealth categories. Key non-migrant members of each community—including local village chiefs, landlords, and local farmers—were also identified and interviewed to provide additional local perspectives on migration and related issues.^4^ These interviews were supplemented by farm visits at all research sites, as well as visits to district assemblies to meet local officials and gather district-level information on in-migration, land tenure, and agro-ecological conditions.

### From cocoa to corn: evolving land use practices and in-migration in Nkoranza South District

This research site, situated in Nkoranza South District, was inhabited largely by migrants from Ghana’s Upper East Region, including Grusi, Frafra, and Kusasi migrants, as well as a minority population of Dagaba migrants from Upper West Region. Additionally, there are migrant settlers from earlier waves of migration from Volta Region and Ashanti Region, who came to the area in the decades following Ghana’s independence in 1957 when it emerged as a site of cocoa production. In 1983, bushfires destroyed the area’s cocoa plantations, and local farmers—along with migrant sharecroppers—began farming mainly food crops, including maize, watermelon, yam, beans, groundnuts, cashew, and cassava. According to qualitative interviews with both long-time migrants and locals, land tenure norms and land use practices have undergone a significant transformation in recent decades at this location. As one local woman remarked regarding land tenure conditions in the 1960s and 1970s, when the area was largely utilized for cocoa production: “Formerly there wasn’t any [land] demarcation: The [village] chief would just show you a piece of land and you would farm up to where your strength could take you [after clearing the forest]” (Nkoranza interview 6). She added that following the bushfires in 1983, many local farmers who had previously engaged in cocoa farming moved out of the immediate area, although they often retained de facto ownership over the land they had previously cultivated by renting it out to migrant tenant farmers from Northern Ghana.

Since the destruction of the area’s cocoa plantations, there has been significant growth in the size of the community—from less than three dozen households in 1983 to at least 200 in 2014—primarily as a result of migration from Ghana’s Upper East Region. One of the key reasons Northern Ghanaian migrants gave for moving to Brong Ahafo was the comparative ease in attaining relatively fertile farmland. As one Kusasi migrant from Upper East Region remarked, “There is scarcity of land [In Upper East Region]. You will not get one acre to farm on!” (Nkoranza interview 23). However, the arrival of migrant tenant farmers from Northern Ghana seeking farmland has contributed to land tenure norms in the area becoming increasingly commercialized. One local farmer who had been in the community for over 60 years remarked, “After the fire, that’s when *abusa* [sharecropping]^5^ started, and some landlords began charging rents from 1985” (Nkoranza interview 1). At the time of my research, the cost of renting land in the community had risen to 50 Ghanaian cedis^6^ an acre, per growing season (or 100 cedis over both major and minor growing seasons). Despite the emergence of the land rental market, some farmers were still engaged in *abusa* arrangements with landlords, while in other cases, farmers paid a bag of maize^7^ per acre to the landowner at the end of each growing season. Additionally, in a small minority of cases, highly successful Northern Ghanaian farmers had effectively purchased tracts of land from local families.

Over time, in-migration had begun to create a degree of land scarcity in the community. According, an elderly Grusi migrant who was one of the only migrants from Upper East Region living in the community prior to the 1980s bushfires:

Now we don’t have any reserve land, whereby we know that a particular area is fertile. We used to move around from this land to that land. Now there is a scarcity of land, and bush fallowing has not been followed. Formerly we would leave a piece of land for two-to-three years and then go back to it, but now there is no land [to accommodate this] (Nkoranza interview 9).

In general, since the opening up of land to Northern Ghanaian migrants after the bushfires in 1983, it is clear that rental agreements based on cash payments have become increasingly common, even as some farmers retain *abusa* arrangements, or pay at the end of the growing season via “acre/bag” (of maize) agreements. As one senior Grusi male migrant commented, when asked if it was still possible for new arrivals to the community to get access to farmland: “Provided you have the cash, you will get land!” (Nkoranza interview 3). This points to a confluence between increased migration, the finite availability of local farmland, and the increasing commercialization of land that is under customary tenure, showing the dynamic relationship between migration, land tenure, and the wider “complex adaptive system” at this case study community.

### Unintended consequences of development: smallholder farming in the shadow of agricultural ventures in Wenchi Municipal District

This research site featured a large population of Northern Ghanaian migrants from Upper West Region—including Dagaba, Sissala, Wala, and Mossi migrants—in part due to the establishment of state-run farms, as well as private plantations, in its vicinity beginning in the 1960s. Although most of these bigger farming operations ultimately failed, owing to the unsuitability of local soils to intensive, mechanized agriculture, they attracted significant numbers of migrant laborers from Northern Ghana, some of whom subsequently became involved in smallholder tenant farming in the area (Amanor 2013). As Amanor (2013) points out, the practices of smallholders in this part of the region—including those of migrants—have undergone substantial shifts in recent decades, with changes in the availability of farm subsidies for chemical fertilizers and the relative decline in soil fertility due to farming using tractors leading many farmers to shift away from growing maize—the dominant crop in the 1970s and 1980s—towards a combination of cassava, groundnuts, and inter-cropped maize. This was also true at the case study community where I conducted research. Additionally, yam—a crop historically dominant in this part of the region—remains the main crop produced by some migrant farmers in the case study community. Some smallholder farmers in this area were also producing tobacco on a contract basis for British American Tobacco, an international conglomerate, until the company ceased its production activities in the area in the 1980s, according to one senior member of the community (Wenchi interview 22).

This part of Brong Ahafo is more arid than the Nkoranza case study site. Additionally, a particular feature of this site is that tenant farmers tend to access land from landlords—typically local families—who reside in nearby village communities. Access to farmland for Dagaba migrants was often achieved via the local land rental market, a process that was often smoothed through migrant social networks, which provided access to landlords via relatives or other relations who could vouch for migrant newcomers. As one second-generation Dagaba male migrant explained,

The lands have different owners, and every owner has their terms: If you don’t agree, you can quit [the land] … You have to go to that community [where they live]. Usually, if you know someone who is already farming on their land, you can go with them (Wenchi interview 8).

Many Muslim migrants, including Sissala, Wala, and Mossi migrants, also acquired land through the rental market in neighboring settlements. There was a wide range in the cost of farmland that was available, according to interview data, varying from between 20 and 50 Ghanaian cedis per growing season (or 40–100 cedis a year, over the major and minor rainy seasons). This depended on both the individual terms of the landlord, as well as the varying suitability of different plots of land for specific crops, with some farmers willing to pay a premium for more fertile land to grow yam, for example. The cost of renting farmland has apparently increased incrementally over recent decades. As one Mossi migrant who had arrived 20 years previously remarked, “It [the rental price] has increased steadily. About once every two years, they [local landlords] will add something small. It went from 6 to 8 to 10 to 15 [cedis per season]” (Wenchi interview 3).

However, some migrants at this site had achieved more favorable terms of access to land through patronage-related gifts of land, financial transactions, or historical family-access arrangements agreed previously by their migrant kin. For example, one way that some Muslim migrants had acquired land was through intermarriage into local Muslim families. As one Mossi migrant remarked, “A man here that I met took me as his son. I married someone from this place, and so that man gave me the land for nothing” (Wenchi interview 2). In the case of another research participant, a Sissala migrant was given as a gift a large tract of 100 acres of land as reciprocation for paying the medical expenses of a local resident (Wenchi interview 9). Some migrants also benefitted from more “traditional” arrangements entered into by relatives in previous decades, which had subsequently been carried over year-on-year. An example of such an arrangement was described by one Dagaba farmer:

When we came here [in 1975] … we were able to get land through [a neighboring village], by paying dues to the chief. … We don’t pay rents on the land, but we give a token to the chief [every year], usually some tubers of yam and 20 cedis (Wenchi interview 21).

In this community, a number of migrant women had established themselves as successful market traders, and were earning significant income from this profession, showing that land access was not the only mediating factor of migrant livelihoods at this case study site. Women also sometimes farmed plots as a form of insurance in cases where their primary income came from running small businesses or market trading, in some cases using their harvests to boost their trading activities.

Overall, the lands accessed by migrants were typically smaller at the Wenchi site than in the other two research sites, reflecting the more fragmented nature of land’s availability in this community. Thus, processes of negotiation over land were taking place against the backdrop of increasing scarcity of good quality farmland. Although in the 1970s this part of the region was relatively sparsely populated, and chiefs made large areas of land available to migrant farmers (see Amanor 2013), more recently acquiring land has become relatively difficult. Thus, a number of migrant settler communities have been established in more remote parts of the district in recent decades where there is less pressure on land, as documented by van der Geest (2011b). As one Dagaba farmer in the case study community put it, “…forever we are tied here. If you go somewhere else and leave the land that you are farming, someone else will come along and take it!” (Wenchi interview 7). In this context, migrant farmers’ social capital formed a key aspect of their relative positionality within the area’s evolving “complex adaptive system”.

### Pru District: cross-river migration for better farming prospects

The majority of the migration from Northern Ghana to this case study site was from the relatively nearby Northern Region, which lies across Lake Volta from Pru District—although the origin communities of migrants were widely dispersed throughout Northern Region. The main migrant ethnic groups in this community include the Gonja, Konkomba, Dagomba, Mamprusi, and Chokossi. Despite the fact that this part of the region is relatively arid in comparison to the other two case study sites, the availability of farmland, including the existence of the seasonal floodplains of Lake Volta (known locally as “the riverside”), offers relatively good farming prospects. Additionally, disputes over customary land ownership in Northern Region have been a key reason for migration to Pru District and are also the main cause of long-running violent conflicts in Northern Region, in particular between the Konkomba, Dagomba, and Gonja. Thus, as Tonah (2007, p. 245) comments, for migrants coming from Northern Region, the farming opportunities in Pru District are comparatively “rosy.” Although Tonah (2007, p. 245) notes that the influx of migrants has led to a relative increase in population density and demand for farmland in the district in recent years, as discussed in the “Research methodology” section, the district has a much lower population density than Nkoranza South and Wenchi Municipal Districts.

In this case study community, there was a nearly universal practice of more “traditional” forms of land tenure for migrants, with tenants typically providing annual yam “tributes” to local landowners or chiefs at harvest time. Pru District’s traditional authority, Yeji Traditional Council, has itself endured an ongoing chieftaincy dispute (see, for details, Ghana National Peace Council 2016) in recent years, and as a result, customary authority over land is relatively fragmented within the district. In this context, migrants in this case study community typically accessed land through several different village chiefs, who exercised control over differing sections of farmland in the vicinity of the community. One Chokossi migrant outlined the typical process through which land was acquired by migrant arrivals:

Before I acquired the land, I presented two bottles of Schnapps to the chief of [the]…village. Then at every festival we also give some yams [as further tribute to the chief] (Pru interview 1).

Additionally, it was common for migrants who followed kin to the community to initially gain access to farmland via farming part of their relatives’ existing plots of land (as was sometimes also practiced in the other case study communities). This was particularly the case for Konkomba and Gonja migrants, who had a longer history of migration to this area than other migrant groups from Northern Region. In the case of some more successful farmers, this initial access to “family land” provided them with a platform to expand to bigger farming plots, in some cases farming multiple plots simultaneously. However, in other cases, farmers with access to family land were merely cultivating small plots that had been fragmented among several family members from one initial kin member’s original access arrangement and thus had limited potential to earn income via their farming activities.

Competing claims to land at this case study site included the presence of Fulani pastoralists who were common in the area, with cattle sometimes destroying tenant farmers’ crops as they moved across the landscape. Despite long-standing (and partly successful) attempts to mediate land use disputes between herders and farmers in the district (see Tonah 2007), my research findings suggest that the destruction of crops by cattle remains an issue among migrant tenant farmers. Additionally, the establishment of two major biofuel plantations in the district in recent years alienated a significant amount of erstwhile farmland from smallholder producers, although this land was previously being used mainly by “local” farmers, rather than migrant tenant farmers, according to local migrants (Pru interview 13).^8^

As with other research sites, the interface between migration and land tenure norms at this site revealed locally evolved social relations between people and land. Land tenure arrangements for migrants retained a more traditional configuration in this case study site in comparison to the other case study communities, possibly due to the district’s relatively low population density, its more remote location in the regional context, the fragmented nature of the local chieftaincy, and the relatively poor quality of the local farmland and rainfall pattern in comparison to other parts of Brong Ahafo. However, this more traditional type of access did not preclude the existence of concurrent processes of large-scale land transactions occurring in the district, with the aforementioned recent establishment of biofuel plantations—via access negotiated by chiefs—being one example of this.

## Analysis of differentiated migrant livelihood trajectories in Brong Ahafo

Qualitative research in the case study sites indicated that at the level of individual migrants, the “success” of their migration was fairly differentiated, according to their own accounts of their current livelihood situations. While the qualitative research was not representative, these distinct trajectories nevertheless offer insights into differentiated outcomes for migrants that are occurring within these communities. Thus, they have implications for theorizing migration’s potential to act as a form of “adaptation” for Northern Ghanaian migrants who have moved out of a relatively marginal environmental region. This section introduces a typology of livelihood trajectories among Northern Ghanaian migrant farmers in Brong Ahafo, based on empirical findings from the three field sites.

The livelihood typology is based on the sustainable livelihoods approach (SLA) (see for example, Scoones 1998). As Scoones (1998, p. 3) observes, SLA consists of asking, “Given a particular *context*... what combination of *livelihood resources* (different types of ‘capital’) result in the ability to follow what combination of *livelihood strategies*…with what *outcomes*?” (emphasis in original). Thus, SLA incorporates analysis of not only access to land (which constitutes a form of “natural” capital within the framework) but also other forms of capital (including human and social capital) that affect rural livelihoods (Scoones 1998, p. 4; see also Scoones 2009). This is because, according to Chambers (1989), poor people in rural areas tend to reduce vulnerability not by maximizing their income but by diversifying their portfolio of assets, which in turn often implies trade-offs between livelihood security and income levels. A key aspect of this framework is an explicit focus on what institutional processes (for example, land tenure systems) mediate different livelihoods pathways (Scoones 1998, p. 3).

Building on SLA’s conceptual foundation, I accounted for the various forms of economic, social, and natural capital possessed by Northern Ghanaian migrants in Brong Ahafo who formed part of my research sample. Analysis of each of these types of capital was based on the following data gathered during my research interviews:

- *Economic capital*: analysis of on-farm and off-farm employment activities undertaken by migrants, as reported during qualitative interviews
- *Natural capital*: size of migrants’ land access agreements with locals, as well as the nature of the tenure terms associated with these agreements
- *Social capital:* migrants’ positionality within their social networks was considered, including their relative seniority, the size of their networks locally, and their ability to access land or other employment pathways via kin links or through investing in relationships with local intermediaries

Based on this analysis, I developed three main livelihood trajectories to account for the livelihoods of migrants, based on their access to the abovementioned types of capital. These trajectories provide only a snapshot of migrants’ livelihood situation at the time of the study, and it is important to acknowledge that some migrants had moved between these different categories over time. Nevertheless, these constructs are useful in capturing the stratified livelihoods that I encountered among migrant tenant farmers from Northern Ghana at the time of the study in 2014 and were constructed in relation to the “climate-migration nexus” debate mentioned in the “Introduction: conceptualizing migration, land tenure, and wider rural transformations as part of a ‘complex adaptive system’” section, regarding whether migration from environmentally marginal areas can be seen as a form of adaptation. Across the three case studies, I classified migrant tenant farmers according to the following typology^9^:

1. Highly adaptive: There was a small minority of migrants who had apparently experienced a genuine transformation of their fortunes since moving to Brong Ahafo. These were highly successful farmers who—through the commercial success of their farming ventures—had been able to significantly increase the acreage of farmland and had also been able to make productive investments that yielded significant non-farm income, for example through building rental properties in nearby towns, starting businesses, investing in livestock, or pursuing their own higher education (or investing in the higher education of their children).
2. Moderately adaptive: By contrast, a substantial number of migrants I interviewed were generally experiencing success through farming or in some cases had established significant off-farm ventures (including a handful of highly successful female market traders at the Wenchi and Pru case study sites). However, in comparison to the “highly adaptive” grouping, many migrants in this group were relatively vulnerable to the seasonal environmental variability, as their reliance on rain-fed agriculture could potentially be undermined by risks posed by rainfall variability, bushfires, and declining soil quality. Nevertheless, these migrants had generally experienced a subjective improvement in their livelihoods as the result of migration and were often providing significant levels of material support for kin in Northern Ghana through sending remittances and/or food crops.
3. Non-adaptive: A final group of interviewees was struggling to eke out a living in Brong Ahafo. These migrants’ farm plots were usually small and they were often just breaking even or, worse, continually “farming at a loss” (as some migrants explained it). They were often in debt to landlords or migrant members of their communities. If they were involved in off-farm work, the income they earned from it was meager. They were particularly vulnerable to seasonal environmental variability as well as economic “shocks” and often were able to provide only small levels of support to kin in the north.

**Fig. 2:**
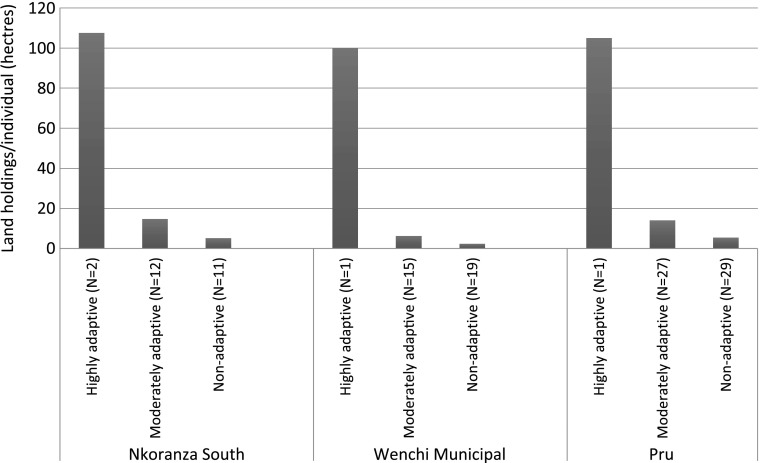
Stratified individual land access (rented and owned) among migrants at the three case study communities: average land holdings by livelihood grouping

In all three of the case study communities, these differentiated livelihood groupings were reflected in migrants’ access to land. Migrants with “highly adaptive” livelihood trajectories, in particular, tended to have much larger land holdings (whether rented or partially owned) than other migrants (see Fig. 2). At the other end of the spectrum, “non-adaptive” migrants often had comparatively very small farming plots, underlining the meager earning potential that they had as tenant farmers in Brong Ahafo in comparison to other migrant tenant farmers. Migrants who were categorized as “moderately adaptive” at each site, meanwhile, usually had more substantial land access than those who were “non-adaptive,” and their livelihood prospects were often also sometimes augmented by some form of off-farm earnings.

There were various local specificities around migrant livelihood trajectories and land access at each site. At the Nkoranza site, those who were “highly adaptive” or “moderately adaptive” tended to have cash rental agreements or own part of their plots, in comparison to those who were “non-adaptive,” who tended to be engaged in sharecropping arrangements. At the Wenchi site, migrants with “highly adaptive” or “moderately adaptive” livelihood trajectories tended to have more favorable land access arrangements (i.e., land that had been given as a gift due to intermarriage or access to “family land” that they shared with other migrant relatives) or fruitful off-farm ventures. By comparison, migrants who had “non-adaptive” livelihoods had smaller farming plots, which they usually accessed through the land rental market and/or more marginal off-farm livelihood activities. At the Pru site, migrants were often able to access comparatively large plots of land, although this was perhaps offset by poorer rainfall and land quality in this part of the region. Migrants with access to greater than ten acres—who were typically classed as having “highly” or “moderately adaptive” livelihood trajectories—had often acquired their land directly from chiefs or other landlords, while those with more marginal livelihoods and in many cases were farming a plot of their relatives’ land.

**Fig. 3:**
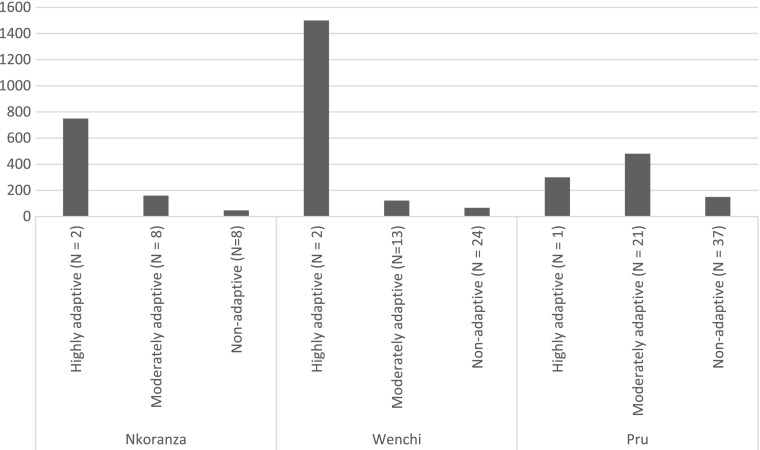
Average annual cash remittances (Ghanaian cedis) sent by Northern Ghanaian migrants, by livelihood trajectory

The differentiated land access for migrants at three case study sites clearly has implications for the extent to which migration to Brong Ahafo can be perceived as a form of “adaptation.” This is illustrated by the amount of cash remittances that migrants sent to kin in Northern Ghana (see Fig. 3 for a summary of this across the three research sites)^10^—which is one instrument for gauging migration’s impact on poverty reduction in communities of origin. While there was not a linear relationship between livelihood trajectories and remittance amounts—as the latter were also affected by the extent to which migrants had less economically well-off relatives who remained in Northern Ghana—the latter nevertheless provide some indication into how the stratified livelihoods of migrants in Brong Ahafo had implications at the other end of the migration chain for kin who have remained behind.

There were differences in remittance behavior across the three case study sites that are important to take into account in this analysis. In Pru District, cash remittances were the main form of support provided to kin in Northern Region, as sending food to relatives via Lake Volta was more expensive than simply sending cash for relatives to buy food or other goods in their home communities. At the Nkoranza site, by contrast, many migrants who sent remittances also sent foodstuffs or other in-kind support, and in some cases migrants (including some of those in the “moderately adaptive” livelihood trajectory) sent food exclusively. At the Wenchi case study site, meanwhile, many of the migrants were already of the second generation. This affected the size of the remittances they sent—which were generally smaller than at the other two sites—owing to the “weaker” nature of kin linkages with relatives in Northern Ghana. Such transfers were often augmented by sending local crops such as maize, yam, or cassava and constituted a small but significant form of ongoing social exchanges with relatives in Northern Ghana.

## Conclusion: migration as adaptation? Land tenure, in-migration, and processes of land fragmentation and accumulation in Brong Ahafo

As Amanor (1994) and van der Geest et al. (2010) have both argued, albeit in different ways, migrants have been drawn to Brong Ahafo’s transition zone in recent decades in part due to the relative abundance of arable land and the lack of heavily commercialized terms of access, which exist elsewhere in Ghana’s forest region. The qualitative findings from the three case study communities presented in preceding sections show that there are multiple configurations of land tenure arrangements between migrant tenant farmers and local hosts operating in the region. I argue that this spectrum of land tenure configurations can be theorized as part of a wider “complex adaptive system”: In the context of significant migration to Brong Ahafo in recent decades, the negotiations around land between migrants and their hosts are just one dimension of a wider set of co-evolving social and environmental processes in the region.

In part, these changes reflect broader processes happening at the regional and national level, including recent large-scale economic reforms. Awumbila and Tsikata (2010, p. 99) argue that since the economic liberalization that accompanied Ghana’s experience of structural adjustment in the 1980s there have been contradictory processes of *concentration* of land in the hands of certain economic interests, as well as *fragmentation* of land holdings, in particular among rural smallholders. This has resulted in “significant inequalities in access to land and its resources” (Awumbila and Tsikata 2010, p. 99). In this vein, customary chiefs have acted as the brokers of large-scale international land deals in recent years, with customary tenure increasingly being interpreted as outright ownership of land in these cases, on par with private property. In the case of Brong Ahafo, there has been major investment in teak, cashew, and exotic mango approved by local chiefs in recent decades (Amanor 2008, pp. 76–77), as well as investment in the biofuel plantations for jatropha (Schoneveld 2013), although in the case of the latter many of these investments have apparently failed.^11^ Such land investment is part of a significant wave of global investment in farmland in countries across the Global South over the past decade, in particular, prompting claims of a new, unprecedented “land grab” (see for example, Schoneveld 2011; Fairhead et al. 2012; Cotula 2013; Scoones et al. 2013; White et al. 2012).

The internal migration of tenant farmers from Northern Ghana to Brong Ahafo interfaces with these larger processes on multiple fronts, with migrants at once creating greater demand for land—in some cases enhancing local perceptions of land’s value—while often retaining a relatively marginal position in local customary land tenure power structures. In a context in which, as Amanor and Pabi (2007) have highlighted, keeping land continuously occupied is one way of expressing de facto land ownership, rental or sharecropping agreements with migrants can help solidify locals’ claims to potentially contested land. At the same time, migrants from Northern Ghana are also moving to Brong Ahafo partly as a result of a structural scarcity of good-quality farmland in *their* communities of origin, as highlighted by van der Geest (2011a), showing that issues regarding land access are vital at both ends of the migratory chain.

In this context, migrants who arrived at destinations in Brong Ahafo when more favorable land tenure norms existed, or who have inherited “family land” under favorable terms from migrant relatives who preceded them, are at a comparative advantage relative to more recent migrant arrivals. While the increasing *double exposure* of smallholders to market and climatic shocks, as theorized by Nyantakyi-Frimpong and Bezner-Kerr (2015), means that land tenure norms are only one dynamic that affects livelihood outcomes for migrant tenant farmers in the rural Ghanaian context, it is nevertheless possible that changing land tenure norms may over time have a wider, amplified effect on the evolving “complex adaptive system” in Brong Ahafo, with respect to migrant tenant farmers’ livelihoods. The relative scarcity of farmland in comparison to past decades as well as changing land tenure norms, which are generally becoming more commercial, may serve as a “feedback” that has a negative impact on the livelihood trajectories of many migrant farmers—especially newcomers. As the differentiated migrant livelihood trajectories presented in the “Analysis of differentiated migrant livelihood trajectories in Brong Ahafo” section suggest, such changes in Brong Ahafo’s “complex adaptive system” have significant implications for theorizing migration to the region from Northern Ghana as a form of adaptation. While for some migrants, migration can evidently be a highly adaptive endeavor, for many others the returns of migration both for themselves and for kin who remain behind in Northern Ghana are relatively negligible.

## Footnotes

1 In this article, “Northern Ghana” refers to Ghana’s three northernmost regions: Northern Region, Upper East Region, and Upper West Region (refer to map in Fig 1).

2 Refer to Moller-Jensen and Knudsen (2008) for a discussion of how this has emerged as a key secondary migration flow in Ghana, according to 2000 census data.

3 The number of migrant interviews conducted at each site was as follows: Nkoranza *N* = 27, Wenchi *N* = 34, and Pru *N* = 60. In Nkoranza, individual migrant interviews were supplemented by focus group duscussions with migrants. More interviews were conducted with migrants in Pru owning to larger number of different ethnic groups residing in this particular site, in an attempt to try to get varied perspectives within each group.

4 NB: The names of research sites and research participants are withheld in this article to protect their anonymity, owing to the “high-risk” nature of this fieldwork, which took place with relatively at-risk internal migrants, according to University of Sussex research ethics guidelines.

5 Under the *abusa* system, tenant farmers give one third of their harvest to the land’s owner.

6 For reference, at time of writing (March 2017), 1 Ghana cedi = .21 US dollars.

7 Worth about 70 Ghanaian cedis, at the time of fieldwork

8 One of these plantations had ceased operation for 18 months prior to my fieldwork, apparently due to financial difficulties, yet the land remained under biofuel cultivation—and thus alienated from potential use by smallholder farmers.

9 The criteria for the classification were as follows: Highly adaptive farmers had been able to greatly expand their land holdings, through rental or other access agreements, and had substantial off-farm earnings through off-farm investments of businesses. Moderately adaptive farmers were typically farmers who had 10–20 acres of land access, with generally successful harvests, or had considerable off-farm earnings (300 cedis a month or greater), as was the case for some female market traders. However, their overall livelihood portfolio was less diverse than those classed as highly adaptive. Non-adaptive migrants had smaller tracts of land, less successful harvests, or were dependent on off-farm income that was meager.

10 Figure 3 includes only cash remittances and does not include other forms of support (including food crops, clothing, and other items) sent by some migrants.

11 George Schoneveld, personal email communication, 2012
